# A Seed Endophytic Bacterium *Cronobacter dublinensis* BC-14 Enhances the Growth and Drought Tolerance of *Echinochloa crus-galli*

**DOI:** 10.3390/microorganisms12122544

**Published:** 2024-12-10

**Authors:** Sheng Cheng, Qingling Wang, Dashan Yang, Quanlong He, Jianxin Deng, Yi Zhou, Lin Zhang, Jianwei Jiang

**Affiliations:** 1 College of Agriculture, Yangtze University, Jingzhou 434025, China; 18972980911@163.com (S.C.); 18695006691@163.com (Q.W.); 13697266606@163.com (D.Y.); 13657161586@163.com (Q.H.); djxin555@hotmail.com (J.D.); zhouyi@yangtzeu.edu.cn (Y.Z.); 2 MARA Key Laboratory of Sustainable Crop Production in the Middle Reaches of the Yangtze River (Co-Construction by Ministry and Province), Yangtze University, Jingzhou 434025, China; 3 Reclamation Foreign Economic Center, Department of Agriculture and Rural of Hubei Province, Wuhan 430071, China

**Keywords:** drought stress tolerance, plant–microbe interaction, plant growth promotion, barnyard grass, seed endophytic bacteria

## Abstract

Successful seed germination and plant seedling growth often require association with endophytic bacteria. Barnyard grass (*Echinochloa crus-galli* (L.) P. Beauv.) is a main weed during rice cultivation and has frequently been found in drought-prone fields such as cornfields in recent years. To determine whether endophytic bacteria enhance the survival chances of barnyard grass in dryland conditions, endophytic bacteria were collected from barnyard grass seeds. An endophytic bacterial strain, BC-14, was selected and confirmed as *Cronobacter dublinensis* based on its morphology, physiology, biochemistry, and genomic information. Moreover, *C. dublinensis* BC-14 secreted IAA in the Luria–Bertani broth up to 28.44 mg/L after 5 days; it could colonize the roots of barnyard grass. After the inoculation with seeds or the well-mixed planting soil, the bacterium can significantly increase the root length and plant height of barnyard grass under drought conditions. When comparing with the control group on the 28th day, it can be seen that the bacterium can significantly increase the contents of chlorophyll b (up to 7.58 times) and proline (37.21%); improve the activities of superoxide dismutase, catalase, and peroxidase (36.90%, 51.51%, and 12.09%, respectively); and reduce the content of malondialdehyde around 25.92%, which are correlated to the drought tolerance. The bacterial genomic annotation revealed that it contains growth-promoting and drought-resistant functional genes. In a word, *C. dublinensis* BC-14 can help barnyard grass suppress drought stress, promote plant growth, and enhance biomass accumulation, which is helpful to interpret the mechanism of weed adaptability in dry environments.

## 1. Introduction

Drought is one of the primary environmental stresses that limit plant growth, and the situation is expected to intensify due to global warming and water scarcity [1,2]. To address this challenge, a variety of mitigation strategies must be implemented. Among these strategies, plant endophytes play a significant role in helping plants to withstand and adapt to drought stress [3]. Beneficial endophytes inhabiting the plant advantageously support plants through various mechanisms against drought stress, including nitrogen fixation, indole-3-acetic acid (IAA) and siderophore production, and phosphate solubilization [4]. In addition, the endophytes induce plants to produce more reactive oxygen species (ROS) scavenging enzymes to survive under drought conditions [5]. Therefore, more drought-tolerant endophytes can be excavated to understand the interaction with their host plants and apply them in drought-resistant cultivation.

In adverse environments, weeds can dominate farmland due to the presence of endophytes that regulate plant growth [6]. Barnyard grass (*Echinochloa crus-galli* (L.) P. Beauv.) is an annual *Poaceae* weed, commonly found in paddy fields but increasingly occurring in maize dry fields in recent years [7,8]. It suggests that barnyard grass has strong adaptability to dry conditions, which probably links to endophytes [9,10]. The application of weed endophytes presents a diverse range of antagonistic forms with short life cycles applied in sustainable agriculture [11]. Beneficial endophytes have been isolated from barnyard grass grown in different stress environments, which can be used for bioremediation of pesticide-contaminated environments [12] and alleviate the adverse effects of salt stress [13] and heavy metals [14] in different plants.

Plant seeds serve as reproductive organs and transmit genetic information, playing a crucial role in agricultural production as well [15]. During reproduction, seeds act as a medium to facilitate the transmission of beneficial endophytes to their offspring, which play an important role in protecting seeds against adverse environments [16]. Those endophytes maintain normal plant growth and development under drought conditions through increasing root and shoot length, plant biomass, leaf area, proline content, sugar, and protein content to improve plant growth, development, quality, and yield [17,18]. An endophytic *Burkholderia vietnamiensis* isolated from *Populus trichocarpa* has been found to promote plant growth through nitrogen fixation and the secretion of indole-3-acetic acid (IAA) [19]. Abdallah et al. [20] reported that endophytic bacteria *Alcaligenes faecalis* from *Withania somnifera* have a high capacity for producing IAA and solubilizing phosphorus, which can enhance the growth of tomatoes while also effectively suppressing *Fusarium* wilt in tomatoes. Endophytic *Pseudomonas* sp. strains OB155 and OS261 have been shown to induce cold tolerance in tomato plants by activating antioxidant capacity in the intercellular spaces of tomato root cells [21]. The endophytic bacterium *Pantoea alhagi* LTYR-11ZT has been shown to promote the growth of wheat and enhance its drought resistance. Strain LTYR-11ZT increases the accumulation of soluble sugars in wheat leaves under drought stress, reduces the accumulation of proline and malondialdehyde (MDA), and decreases chlorophyll degradation [22]. Seed endophytes are valuable resources for agricultural production [23], but there is little research on barnyard grass seed endophytes and their properties in improving the drought tolerance for plants. Whether seed endophytic bacteria could enhance the survival chances of barnyard grass in dryland conditions still needs to be confirmed. This study aims to select a plant-promoting bacterial candidate from barnyard grass seeds collected from drought fields, to identify the bacterium based on cultural, molecular, and biochemical characterization, and to evaluate and analyze the abilities to improve the drought tolerance for barnyard grass. It may provide an important reference for applying seed endophytic bacteria against drought stress in agriculture.

## 2. Materials and Methods

### 2.1. Seed Collection and Endophytic Bacterial Isolation

Seeds of barnyard grass were collected from drought fields of Mapaoquan Village (39°91′ N; 116°41′ E), Jingzhou City, Hubei Province, China, in September of 2023. The area is characterized by barren slopes, with high terrain and distance from water sources, making irrigation impossible, and plants are perpetually in a state of water deficiency. During seed collection, the vegetation was experiencing drought conditions. Healthy seeds were obtained and stored in clean kraft paper bags at 4 °C until use. The seed surface sterilization method was carried out according to Kumar et al. [24], and 100 μL of the final rinse sterile water was spread on Luria–Bertani agar (LA) medium to evaluate the success of the methodology [25]. The surface-sterilized seeds were ground in a sterilized mortar with 5 mL of sterilized distilled water. The ground supernatant was diluted to 10^−4^ to 10^−7^ and spread on LA medium. After incubation at 28 °C for 3–5 days, single colonies were selected for purification based on their shape, color, size, and other traits. The purified strains were stored in glycerol suspensions (20%, *v*/*v*) at −80 °C.

### 2.2. Plant Growth Promotion

The seed endophytic bacteria were individually cultured in 50 mL of LB medium in a rotary shaker with 150 rpm rotation for 48 h at room temperature. The cultural broth was centrifuged at 8000 rpm for 10 min. The bacteria were resuspended in sterile water and adjusted to approximately 10^8^ CFU/mL using a spectrophotometer (BIO-RAD, Hercules, CA, USA) for further use as bacterial inoculum [26]. Surface-sterilized barnyard grass seeds (*n* = 100) were immersed in the bacterial solution for 1 h and placed on moistened paper filter papers in Petri dishes [27]. The seed germination rate was recorded after one day. Then, the germinated seeds were taken off, and 8 healthy germinated seeds were randomly retained per dish (*n* = 3) for continued incubation. After 5 days, the root and leaf length, fresh weight, and dry weight (85 °C for 48 h to constant weight) of seedlings were examined. The experiment was repeated three times to find a great candidate for further study.

### 2.3. Identification of C. dublinensis BC-14

The morphology, size, and surface characteristics of the colony were determined on LB agar plates at 28 °C for 24 h. Gram staining was conducted to observe the bacterial shape and color using a Nikon ECLIPSE Ni-U microscope system (1000×, Nikon, Tokyo, Japan). Biochemical investigation was performed by the Gram-negative bacteria identification kit following the instructions (BioMérieux, Marcy-l’Étoile, France). The genomic DNA was extracted using the FastPure Bacteria DNA Isolation Mini Kit (Vazyme Biotech Co., Ltd., Nanjing, China). For nucleotide sequencing, the partial region of the 16S rRNA gene was amplified using primers 27F/1492R [28]. The PCR products were purified and sequenced by BGI company (Shenzhen, China). The obtained sequences were deposited in GenBank, and the relevant sequences were retrieved from the GenBank database for the sequence analysis. All the sequences were aligned and edited in MEGA v.7.0 software [29]. The maximum likelihood (ML) analysis was conducted using RAxML v.7.2.8 [30] and assessed by bootstrapping with 1000 replicates. Additionally, the genome of the strain BC-14 was accomplished by BENAGEN (Wuhan, China) using the Illumina MiSeq platform. Sequence read quality control and trimming were performed using FastQC v0.11.8 [31] and fastp v0.20.0 [32]. De novo sequence assembly was performed using the program Unicycler 0.4.8 [33]. The assembled genome sequence was analyzed by FastANI 1.3 [34] and GGDC (https://ggdc.dsmz.de/ggdc.php (accessed on 21 August 2024)) to calculate the average nucleotide identity (ANI) and DNA–DNA hybridization (DDH) values. The calculation results are visualized through the pheatmap package 1.0.12 in R 4.4.0 [35]. The genome sequences underwent gene prediction using Prokka 1.14.6 [36], employing the default parameters. Single-copy orthologous genes from *Cronobacter* spp. genomes were identified and analyzed using OrthoFinder 2.5.5 with default settings [37]. The resulting sequences were aligned using MAFFT v7.525 [38], and non-informative columns from the concatenated alignment were trimmed with trimAI v1.5.rev0 [39]. A phylogenetic tree was then constructed using RAxML v7.2.8 [30] and visualized with Figtree 1.4.4 (http://tree.bio.ed.ac.uk/software/figtree (accessed on 22 August 2024)). The genome sequences were subjected to BLAST analysis with COG, KEGG, Swiss-Prot, and RefSeq databases, which yielded a series of gene function annotations related to regulating plants resistant to drought stress [36].

### 2.4. Indole Acetic Acid (IAA) Production of C. dublinensis BC-14

To determine the IAA production secreted by the strain BC-14 according to Cheng et al. [40]. The bacterial solution (1 mL, 10^8^ CFU/mL) was inoculated into LB medium (50 mL, containing 10 g/L L-tryptophan as a precursor) for seven days (*n* = 3). Every day, the cultural broth was sampled and centrifuged, from which the supernatant (2 mL) was collected and added to the same volume of Salkowski’s reagent to incubate at room temperature in the dark for 30 min. The emergence of the red color indicated the production of IAA, which was quantified using an ultraviolet spectrophotometer through the standard curve of IAA concentrations at 530 nm [15]. The experiment was conducted three times.

### 2.5. Phosphate-Solubilizing and Siderophore Production of C. dublinensis BC-14

The plate-based assays were employed to assess the phosphate solubilization and siderophore production of *C. dublinensis* BC-14. To test the phosphate solubilization, the National Botanical Research Institute’s phosphate growth liquid medium (NBRIP) was used [41]. Siderophore production was determined using Chrome azurol S (CAS) agar plates [42]. The bacterial inoculums were inoculated onto the agar media and incubated at 28 °C. The diameters of the halos surrounding the colonies were measured to evaluate both assays after 7 days. The experiment was conducted three times.

### 2.6. GFP-Labeled C. Dublinensis BC-14 and Endophytic Establishment Studies

The GFP-labeled BC-14 strain was constructed by using the plasmid in Liang et al. [43] through the conjugation transfer method. Surface-sterilized barnyard grass seeds were immersed in the bacterial inoculum (10^8^ CFU/mL) for 1 h and subsequently incubated on moistened filter papers in Petri dishes. The seedlings were collected and rinsed thoroughly with sterile water after 7 days. Root sections were obtained using a cryostat microtome (Leica CM1860UV, Nussloch, Germany). All the sections were visualized using confocal microscopy (Leica DMi8, Nussloch, Germany) at an excitation wavelength of 490 nm and an emission wavelength of 510 nm.

### 2.7. Pot Assay for Barnyard Grass Applied with C. dublinensis BC-14 Under Drought Stress

For the pot tests (pot diameter: 10 cm, height: 10 cm), the soil was autoclaved at first and then individually well-mixed with distilled water or the bacterial inoculum (BC-14, 10^8^ CFU/mL) with the same amount. The soil was filled to a height of 8 cm in the pots. The surface-sterilized seeds of barnyard grass were soaked for 2 h using water or the bacterial inoculum and then sowed in pots (*n* = 3) according to the different treatments. Then, the soil was irrigated with 0% PEG and 20% PEG solutions to simulate normal environments and drought stress, respectively. Hence, the experimental treatments were determined as follows: Blank control (C−): The seeds were soaked in sterile water and then sown in aseptic soil. Soil bacteriome (C+): The seeds were soaked in sterile water and then sown in bacterial (BC-14) mixed soil. Seed bacteriome (S−): The seeds were soaked in the bacterial inoculum (BC-14, 10^8^ CFU/mL) and then sown in aseptic soil. The plants were grown in a greenhouse with the routine light period (12-h light/dark period) at room temperature for 28 days. The root length and plant height were determined every week to evaluate the growth of barnyard grass under drought stress. In addition, on the 7th, 14th, 21st, and 28th days after treatment, leaves were collected from the same position on the plants. Subsequently, the instructions of the Solarbio Assay Kits (Solarbio, Beijing, China) were followed (kit codes: BC0990, BC0290, BC0020, BC0170, BC0090, and BC0200); the activity of chlorophyll (total Chl, Chl a, and Chl b), proline (Pro), and malondialdehyde (MDA) in the leaves, as well as the activities of superoxide dismutase (SOD), peroxidase (POD), and catalase (CAT), were extracted and measured.

### 2.8. Statistical Analysis

Data were analyzed using SPSS 26.0 software (SPSS Inc., Chicago, IL, USA) with one-way ANOVA to assess the variability and validity of the results. Duncan’s multiple range test was performed to determine the significance between the treatment groups at *p* ≤ 0.05.

## 3. Results

### 3.1. Selection of Endophytic C. dublinensis BC-14 from Barnyard Grass Seeds

**Figure 1:**
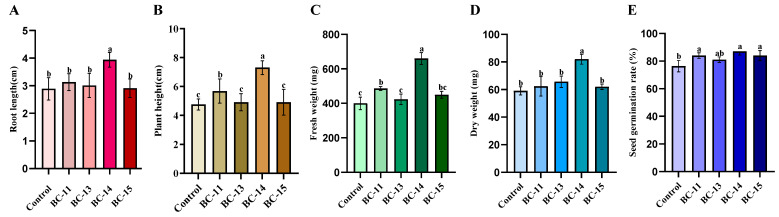
The effects on barnyard grass ((**A**) root length; (**B**) leaf length; (**C**) fresh weight; (**D**) dry weight; and (**E**) seed germination rate) applied with four endophytic bacteria. There are significant differences between data groups represented by different lowercase letters (*p* < 0.05).

Among 298 endophytic bacteria isolated from barnyard grass seeds, 19 candidate bacteria numbered BC-1 to BC-19 were selected for growth promotion tests (Table S1). Four strains exhibited significant growth-promoting impacts and were repeated in the test to ensure the stability of these effects (Figure 1). The strain BC-14 exhibited great activity in promoting the growth of barnyard grass with stable effectiveness, which significantly increased the average root length (3.94 cm), plant height (6.96 cm), fresh weight (660.00 mg), dry weight (79.67 mg), and seed germination rate (87%) compared with the control and other bacteria (Figure 1). Therefore, the strain BC-14 was selected for further research.

### 3.2. Identification of C. dublinensis BC-14

**Figure 2:**
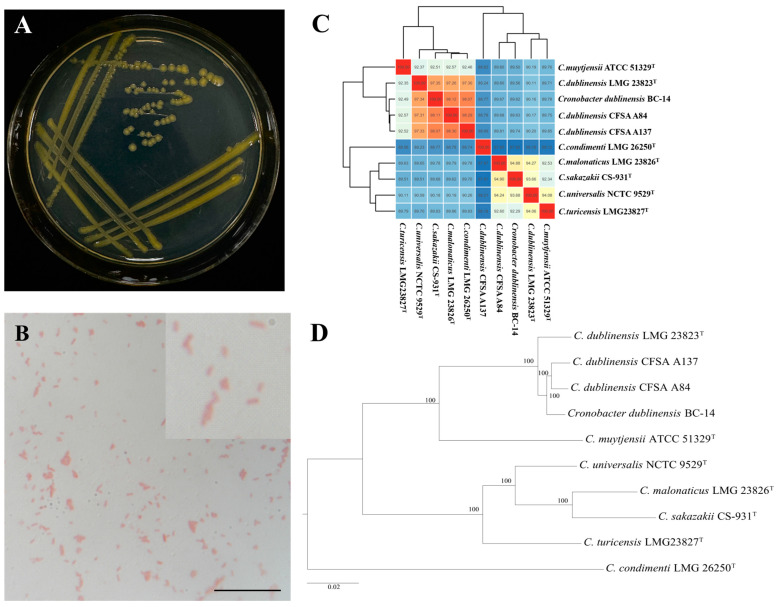
Identification of *Cronobacter dublinensis* BC-14. (**A**) Cultural characteristics. (**B**) Gram staining. (**C**) Heatmap of ANI analysis based on the genome sequences of 10 *Cronobacter* strains. (**D**) The phylogenetic tree based on single-copy ortholog gene sequences of genomes using the maximum likelihood (ML) method. Bootstrap values from 1000 replicates are shown at nodes. *Cronobacter condimenti* LMG 26250 was selected as the outgroup. ^T^: type strain. Scale bar: B = 25 μm.

The BC-14 colonies grown on LA medium were uplifted yellow circles with smooth and complete surfaces. After 5–7 days, the color became lighter and more transparent. The Gram-stained test was red and short rod-shaped, indicating that it was a Gram-negative bacterium (Figure 2A,B). A phylogenetic tree based on the 16S rRNA gene region was constructed containing 18 strains of closely related *Cronobacter* species (Figure S1). In the phylogenetic analysis, the present strain (accession no. PQ533183) fell into a clade together with two strains of *C. dublinensis* supported by 95% bootstrap values (Figure S1). Moreover, the strain BC-14 was also verified by genomic sequencing (accession no. JBIPQX000000000), containing 4,491,274 base pairs with a GC content of 58.09% (Table 1). The ANI (Average Nucleotide Identity, ANI) and DDH (DNA–DNA hybridization) values were, respectively, 97.34% (*C. dublinensis* LMG 23823T) and 87.9% (*C. dublinensis* LMG 23823T) (Figure 2C and Table 1). Based on the phylogram generated with single-copy ortholog sequences of genomes, the bacterium BC-14 was further diagnosed as *C. dublinensis* with a bootstrap value of 100%. (Figure 2D). The biochemical characteristics of *C. dublinensis* BC-14 tested by a Gram-negative bacteria identification kit showed the arginine dehydrogenase, lysine decarboxylase, ornithine decarboxylation, citric acid, urease, gelatin, sorbitol, rhamnose, sucrose, melibiose, mannitol, glucose, amygdalin, and arabinose reactions were positive. H_2_S production, β-galactosidase, and inositol reaction were negative (Table 2).

### 3.3. Functional Detection of C. dublinensis BC-14 and Its Colonization

**Figure 3:**
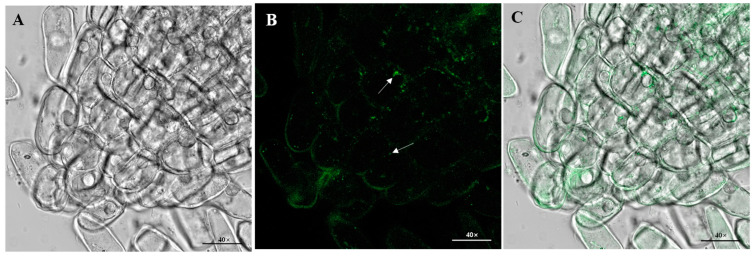
GFP-tagged *Cronobacter dublinensis* BC-14 localization in barnyard grass roots using confocal laser microscopic images ((**A**) white light; (**B**) fluorescence mode; and (**C**) overlay image). The white arrows represented bacteria colonizing the roots.

The bacterium could secrete IAA in LB medium because it showed positive results after being determined with Salkowski’s reagent (Figure S2A). Additionally, the quantitative analysis indicated that it could produce the highest concentration of IAA on the fifth day, peaking at 28.44 ± 1.74 mg/L; the secreting amount exhibited a temporal increase, followed by a subsequent decline after 5 days (Figure S2B). Plate-based assays showed the halos around the colonies, indicating that the strain BC-14 could dissolve phosphorus and produce siderophores (Figure S3). The roots of barnyard grass inoculated with GFP-tagged *C. dublinensis* BC-14 presented the bacteria in root tip cells (Figure 3) observed under the confocal microscope. The results indicated that the bacterium was capable of establishing itself as an endophyte in barnyard grass roots.

### 3.4. Pot Study on Drought Stress

**Figure 4:**
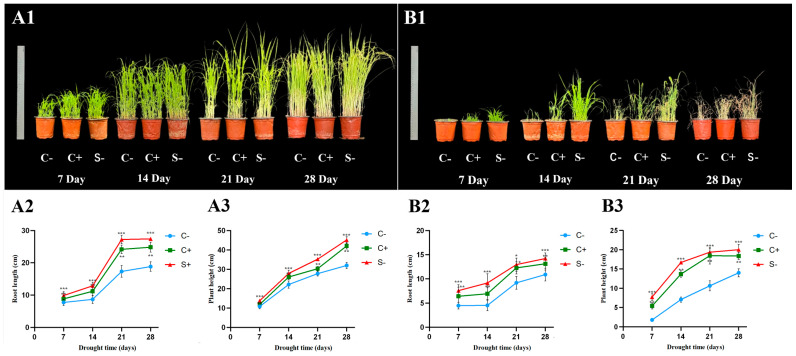
The growth appearance (**A1**,**B1**), root length (**A2**,**B2**), and plant height (**A3**,**B3**) of barnyard grass treated with *Cronobacter dublinensis* BC-14 without (A: 0% PEG) and under (B: 20% PEG) drought stress conditions at 7, 14, 21, and 28 days. C−: The seeds were soaked in sterile water and then sown in aseptic soil; C+: The seeds were soaked in sterile water and then sown in bacterial (BC-14) mixed soil; S−: The seeds were soaked in the bacterial inoculum (BC-14, 10^8^ CFU/mL) and then sown in aseptic soil. ***: significant difference compared with C− and C+, *p* < 0.05; **: significant difference compared with C− and S−, *p* < 0.05; *: significant difference compared with C, *p* < 0.05. The same figure below.

The pot study demonstrated that barnyard grass treated with *C. dublinensis* BC-14 (C+ and S−) showed great plant growth-promoting abilities without drought stress (0% PEG), but it was significant in increasing plant growth and improving the drought tolerance of barnyard grass, compared with their control group (C−) (Figure 4). Especially, the control barnyard grass completely withered, while *C. dublinensis* BC-14 inoculated barnyard grass was still partially alive under the drought stress (20% PEG) (Figure 4B1). The overall plant growth (root length and plant height) of the S− group was the greatest, followed by C+ and C−, either of the treatments (Figure 4). In the treatment without drought stress, the root length and plant height of barnyard grass treated with *C. dublinensis* BC-14 were increased by 14.20%—57.24% and 9.63%—41.15%, respectively, in comparison with the controls (Figure 4A1–A3); however, it was significantly improved by 20.57% to 102.45% and 31.12% to 334.10% under the drought stress condition, respectively (Figure 4B1–B3).

**Figure 5:**
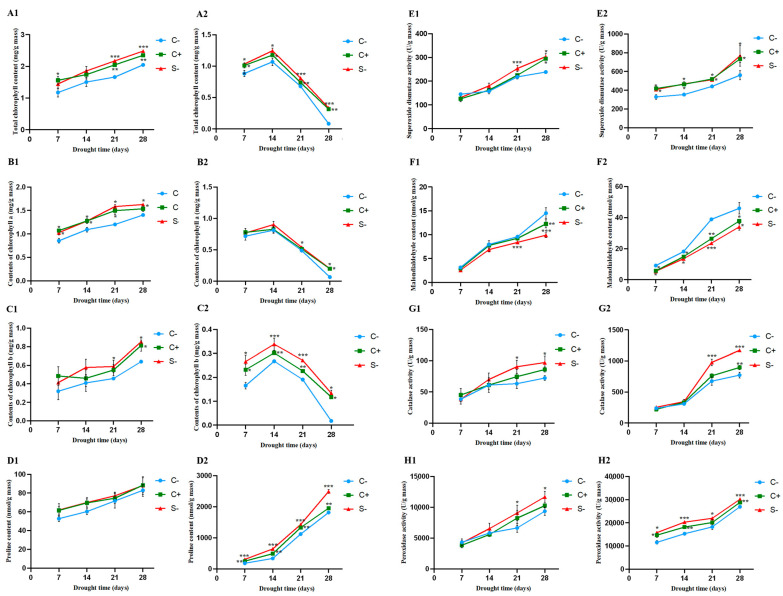
The variations of chlorophyll (**A1**,**A2**), chlorophyll a (**B1**,**B2**), chlorophyll b (**C1**,**C2**), proline (**D1**,**D2**), superoxide dismutase (**E1**,**E2**), malondialdehyde (**F1**,**F2**), catalase (**G1**,**G2**), and peroxidase (**H1**,**H2**) in barnyard grass at 7, 14, 21, and 28 days after the treatment with *Cronobacter dublinensis* BC-14 without ((**A1**–**H1**): 0% PEG) and under ((**A2**–**H2**): 20% PEG) drought stress conditions.

The variations of the total Chl, Chl a, and Chl b, Pro, MDA, SOD, CAT, and POD in barnyard grass at 7, 14, 21, and 28 days were consistent with the plant growth results after the treatment with *C. dublinensis* BC-14 without and under drought stress (Figure 5). Without drought stress treatment (Figure 5A1–H1), the contents of Chl b and Pro and the activities of SOD, CAT, and POD in the S− and C+ groups were not significantly different from those in the C− group before 14 days but significantly higher after 21 days. Exceptionally, the content of Chl a in S− and C+ groups was significantly higher than in controls. Under 20% PEG drought stress (Figure 5A2–H2), the contents of Chl b and Pro, and the activities of SOD, CAT, and POD in the S− group were significantly increased compared to those of the C− group, especially after 21 days. When compared to the C−, the Chl b content of the S− group increased 6.58 times, and 37.21% of Pro; the activity of SOD increased 36.90%, 51.51% of CAT, and 12.09% of POD; the content of MDA decreased 25.92% (Figure 5). In summary, barnyard grass treated with *C. dublinensis* BC-14 (S− and C+) can remarkably increase the Chl content and antioxidant enzyme activity and reduce the MDA content by comparison with the C− group, while the S− group displayed a better effect than the C+ group.

### 3.5. Genome Sequencing and Annotation

**Figure 6:**
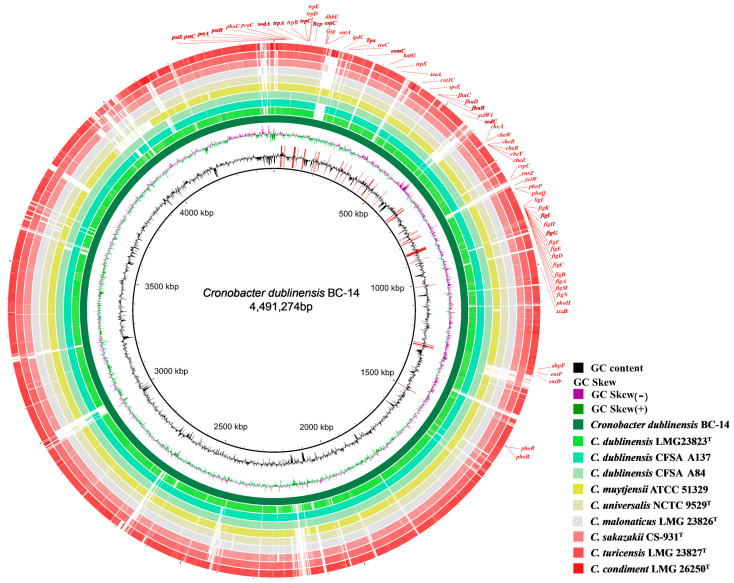
The whole genome sequence of *Cronobacter dublinensis* BC-14.

Genomic analysis revealed that *C. dublinensis* BC-14 encompassed 4236 genes, including 4058 coding genes, 71 tRNA genes, and 4 genomic islands. Additional gene predictions are also outlined (Table S2). Further genomic functional genes associated with plant growth and drought-resistant abilities (IAA production, phosphate solubilization, siderophore production, root colonization, oxidative stress, etc.) were annotated (Figure 6 and Table S3).

## 4. Discussion

Drought stress is one of the most damaging abiotic stresses that has become more intense in recent decades and leads to a reduction in plant growth, development, and production [44], by which the plant-promoting microbes possess the capability to help crops combat drought. Plant growth-promoting bacteria possess various mechanisms to enhance plant drought tolerance, mainly accomplished by secreting IAA, siderophores, spermidine, and trehalose, and dissolving phosphorus and potassium [45]. For example, *Sphingomonas* sp. Cra20 has been reported to preserve genes related to the production of IAA, spermidine, and trehalose genes in its genome to enhance drought resistance and promote growth for *Arabidopsis thaliana* [46]. *Bacillus ayabatensis* NC285 was isolated from oil tea Camellia with the ability to dissolve inorganic phosphorus [47]. *Pseudomonas* strain LTGT-11-2Z can promote the drought resistance of wheat during incubation, and its genome has the siderophore synthesis genes [48]. In this study, the Salkowski colorimetric technique and plate-based assays showed that *C. dublinensis* BC-14 could produce IAA (Figure S1) and phosphate solubilization and secreting siderophores (Figure S2). The genomic analysis (Table S3) showed the evidence in gene level by harboring the related genes for IAA (trpABCDES and ipdC), spermidine (Gsp and speE), and trehalose (TreAC) production, and for siderophores (entACDF) and the dissolution of inorganic phosphorus (phoBHOPUR). These consequences of numerous *Cronobacter* strains, such as *C. sakazakii*, *C. dublinensis*, *C. muytjensii*, and *C. turicensis*, have shown IAA-producing, phosphate-solubilizing, and siderophore-secreting abilities [49,50,51]. In addition, the bacterial cold shock protein genes remain in the genome of BC-14, which were reported to enhance the drought tolerance of maize and wheat [52]. These characteristics of *C. dublinensis* BC-14 may promote the root length and plant height and improve the drought tolerance of barnyard grass.

The failure of beneficial bacteria used in the field may be attributed to their frustrated colonization within plants [53]. Colonization is regarded as one of the essential prerequisites influencing the effectiveness of the ability of endophytes to promote plant growth [54]. It was previously reported that many bacteria can colonize different plants as endophytes [55], such as *Bacillus thuringiensis* RZ2MS9, which can promote the growth of maize by colonizing the roots and leaves [56], *Paenibacillus polymyxa* P2b-2R increasing the length of canola seedlings by 70% after colonization [57], and *C. dublinensis* alleviating the adverse effects of drought stress for pearl millet by the existence in the roots [58]. *Cronobacter* spp. can live as epiphytic and endophytic microbes in the roots of tomato and maize, beneficial to plant growth [51]. In addition, *Cronobacter* species have attracted attention because they can promote plant growth and reduce the impact of environmental stress on plants [59,60]. *Cronobacter muytjensii* JZ38 can promote *Arabidopsis thaliana* growth under salt stress by contacting and releasing volatile compounds [59], and *Cronobacter* sp. Y501 can enhance the growth of maize and improve its drought resistance [60]. *Cronobacter dublinensis* is first reported to be preserved in seeds of barnyard grass and to colonize its roots in this study, which guarantees its ability to promote barnyard grass growth and resist drought conditions. Furthermore, its genome encodes flagellar assembly and chemotaxis-related proteins, which may contribute to plant adhesion. The findings enrich the resources of beneficial plant endophytic microorganisms and drought-resistant microbial resources.

Drought stress affects photosynthesis and chlorophyll synthesis and produces excessive reactive oxygen species (ROS) and MDA [61,62]. In plants, higher levels of ROS can cause membrane degradation, lipid peroxidation, and the degradation of nucleic acids, proteins, and lipids [63], which can result in significant damage to the chloroplast membrane system in leaf cells, adversely affecting normal plant growth. The interaction between microorganisms and plants is crucial for coping with climate change [64], affecting the growth, development, and adaptation in extreme environments. The experimental results indicate that compared to the control group, the significant increase in chlorophyll content in the S− (seed bacteriome) and C+ (soil bacteriome) groups suggests that *C. dublinensis* BC-14 plays a role in maintaining or improving the mechanisms of photosynthesis (Figure 5A1–C1,A2–C2), which is crucial for the survival of plants under drought conditions [65]. Inoculation with *Bacillus pumilus* under drought stress improves the integrity of chloroplast and mitochondrial cellular structures, increases chlorophyll content, photosynthetic parameters, and water use efficiency, promoting the growth and drought resistance of *Glycyrrhiza uralensis* [66]. Similarly, the current results show that *C. dublinensis* BC-14 could also significantly increase the proline content and the activities of SOD, CAT, and POD in barnyard grass under both normal and drought stress conditions (Figure 5D1–H1,D2–H2). The enhancement of these antioxidant defense systems is essential for scavenging reactive oxygen species (ROS) and alleviating oxidative damage [67]. Proline, as an osmoprotectant, helps stabilize proteins and cellular structures under conditions of water scarcity [68], increasing drought resistance. Inoculation of maize with *Bacillus* sp. under drought stress can increase proline content to combat drought [69]. Research has found that *Burkholderia phytofirmans* enhances the tolerance of *Arabidopsis* to drought and salt stress by increasing the activity of antioxidant enzymes and reducing the content of MDA [70]. The significant increase in these enzyme activities, especially after 21 days, indicates that *C. dublinensis* BC-14 may induce long-term adaptations in plant antioxidant mechanisms, providing sustained antioxidant protection [71]. Plants also activate a series of defense mechanisms when infected by pathogens [72]. For example, infection by *F. culmorum* increases the activity of CAT and POD, as well as the concentration of phenolic compounds in wheat, to resist the pathogen [73]. However, endophytes enhance plant health and productivity by promoting plant growth and stress resistance [74], while the defense responses triggered by pathogens, although helpful in resisting the pathogens, often come at the cost of growth [75]. Additionally, the S− group (seed bacteriome) is more effective than the C+ group (soil bacteriome) in helping barnyard grass resist drought stress, possibly due to direct early contact with the seeds (Figure 4). This early colonization may activate plant defense mechanisms, providing an advantage in dealing with environmental stress [76]. These results may explain the frequent occurrence of barnyard grass in drylands with the help of its endophytes.

## 5. Conclusions

*Cronobacter dublinensis* BC-14 was isolated and selected from barnyard grass seeds on drought fields, and it can colonize the roots to improve the growth and drought stress tolerance of barnyard grass. Pot study showed that BC-14 could increase the content of chlorophyll and proline, increase the activity of ROS scavenging enzyme, and decrease the content of MDA under normal and drought stress. In addition, *C. dublinensis* BC-14 promoted the growth of barnyard grass by producing IAA and siderophores and solubilizing phosphorus. *C. dublinensis* BC-14 enhances barnyard grass drought tolerance and growth by modulating stress-responsive compounds and promoting nutrient availability.

## Figures and Tables

**Table 1:** Raw data: information of *Cronobacter* strains for phylogenetic analysis.

Species	Strain ID	Source	Location	Accession Numbers	Base Size (bp)	Scaffold N50	No. of Scaffolds	Percent G + C (%)	No. of Genes	No. Proteins	No. Plasmids	Plasmid Size (bp)	ANI with BC-14 (%)	DDH with BC-14 (%)
*C. condimenti*	LMG 26250^T^	spiced meat	Slovakia	GCF_001277255.1	4,499,482	4,347,991	2	56	4242	4059	1	164,790	88.77	80.8
*C. dublinensis*	BC-14	barnyard grass	China	JBIPQX000000000.1	4,491,274	805,486	21	58.09	4236	4058	1	151,085	100	100
*C. dublinensis*	LMG 23823^T^	milk powder plant	Ireland	GCF_001277235.1	4,628,405	4,431,067	2	58	4379	4224	1	203,534	97.34	87.9
*C. dublinensis*	CFSA A137	noodle	China	GCF_032598745.1	4,768,277	4,532,538	3	57.5	4590	4315	2	174,783/60,956	98.07	89.7
*C. dublinensis*	CFSA A84	noodle	China	GCF_029227785.1	4,733,612	4,526,300	4	57.5	4561	4347	3	162,169/38,910/6233	98.12	91.6
*C. malonaticus*	LMG 23826^T^	breast abscess	America	GCF_001277215.2	4,473,761	4,294,639	3	57	4292	4090	2	126,501/52,758	89.67	78.7
*C. muytjensii*	ATCC 51329^T^	-	France	GCF_001277195.1	4,364,114	4,364,114	1	57.5	4100	3934	0	0	92.49	81.6
*C. sakazakii*	CS-931^T^	feces	Mexico	GCF_003516125.1	4,437,993	4,267,208	3	57	4228	4050	2	119,197/51,588	89.62	76.5
*C. turicensis*	LMG23827^T^	blood culture	Switzerland	GCF_041222865.12	4,554,702	4,384,662	3	57.5	4329	4137	2	147,683/22,357	89.78	83.2
*C. universalis*	NCTC 9529^T^	water	-	GCF_001277175.1	4,436,873	4,307,096	2	57	4210	4039	1	136,454	90.16	80.3

Abbreviation: ^T^ = type strain.

**Table 2:** Biochemical characteristics of *Cronobacter dublinensis* BC-14.

Characteristic	Result	Characteristic	Result	Characteristic	Result
β-galactosidase	−	Urease	+	Mannitol oxidation	+
Arginine dehydrogenase	+	Gelatin liquefaction	+	Inositol oxidation	−
Lysine decarboxylase	+	Sorbitol oxidation	+	Glucose oxidase	+
Ornithine decarboxylation	+	Rhamnose oxidation	+	Amygdalin oxidation	+
Citric acid utilization	+	Sucrose oxidation	+	Arabinose oxidation	+
H_2_S production	−	Melibiose oxidation	+		

“+” denotes positive results; “−” denotes negative results.

## Data Availability

The novel findings presented in this study are included in the article/Supplementary Materials; any queries can be addressed to the corresponding authors. The BC-14 genome data presented in this study are available in the National Centre for Biotechnology Information (NCBI) database (BioSample: SAMN43986877 at the NCBI database.).

## Supplementary Materials

The following supporting information can be downloaded at: https://www.mdpi.com/article/10.3390/microorganisms12122544/s1. Table S1. Activity-promoting effect of endophytic bacteria on barnyard grass seeds (preliminary screening). The different lowercase letters in the table show significant differences (*p* < 0.05), and the following table is the same as above; Table S2. Genome properties of *Cronobacter dublinensis* BC-14; Table S3. Functional genes associated with plant growth and drought resistance for *Cronobacter dublinensis* BC-14; Figure S1. The phylogenetic tree based on 16S rRNA using the maximum likelihood method. Bootstrap values (>60%) from 1000 replicates are shown at nodes. *E. coli* NBRC 10220 was selected as the outgroup. The present strains are marked in bold. ^T^ = type strain; Figure S2. Detection of the IAA production in *Cronobacter dublinensis* BC-14. (A) Salkowski test of the negative control (left) and the *Cronobacter dublinensis* BC-14 broth (right). (B) The IAA concentration in different incubation times; Figure S3. Clear zone formation around the colony in inorganic phosphorus agar medium indicated the ability to solubilize inorganic phosphorus (A); yellow-orange halos in CAS agar medium indicated siderophore production (B).
