# Supplementary material for: A Seed Endophytic Bacterium Cronobacter dublinensis BC-14 Enhances the Growth and Drought Tolerance of Echinochloa crus-galli
Source: Microorganisms. 2024 Dec 10;12(12):2544. doi: 10.3390/microorganisms12122544 (PMC11677215; doi:10.3390/microorganisms12122544)
Supplement: Supplementary file 1 [file microorganisms-12-02544-s001.zip › microorganisms-3338582-supplementary.pdf]

Tabel S1 Activity-promoting effect of endophytic bacteria on barnyard grass seeds (preliminary screening). The different lowercase letters in the table show significant differences ($P < 0.05$), and the following table is the same as above.

Strain name	Root length	Plant height	Fresh weight	Dry weight	Seed germination rate
Control	$3.10±0.34^{def}$	$4.91±0.41^{cde}$	$434.00±8.19^{bcd}$	$62.33±2.08^{de}$	$77.33±2.52^{d}$
BC-1	$3.25±0.64^{bcde}$	$4.61±0.58^{de}$	$424.33±8.62^{cd}$	$64.67±1.53^{cde}$	$81.33±2.52^{bcd}$
BC-2	$3.21±0.32^{cde}$	$4.55±0.53^{def}$	$426.33±16.65^{cd}$	$61.67±1.15^{def}$	$78.00±2.65^{d}$
BC-3	$2.67±0.41^{gh}$	$4.79±0.61^{cde}$	$428.00±12.12^{cd}$	$63.33±1.53^{de}$	$82.33±2.08^{abcd}$
BC-4	$2.52±0.29^{h}$	$4.07±0.50^{fg}$	$394.67±8.50^{ef}$	$53.00±4.36^{g}$	$81.00±2.65^{bcd}$
BC-5	$3.17±0.44^{de}$	$3.96±0.46^{g}$	$373.67±8.96^{fg}$	$51.67±3.21^{g}$	$78.00±4.58^{d}$
BC-6	$3.23±0.38^{cde}$	$4.58±0.69^{de}$	$426.67±4.93^{cd}$	$61.00±2.00^{ef}$	$80.67±1.53^{bcd}$
BC-7	$3.13±0.64^{def}$	$5.02±0.87^{cd}$	$433.67±6.81^{bcd}$	$67.33±2.08^{cd}$	$76.67±4.04^{d}$
BC-8	$3.05±0.47^{def}$	$4.49±0.69^{ef}$	$414.33±8.33^{cde}$	$63.33±2.52^{de}$	$78.00±3.00^{d}$
BC-9	$2.79±0.27^{fgh}$	$3.81±0.40^{g}$	$357.33±5.69^{gh}$	$53.00±2.00^{g}$	$78.67±4.93^{cd}$
BC-10	$3.56±0.54^{abc}$	$3.84±0.45^{g}$	$344.67±27.74^{h}$	$56.00±3.00^{fg}$	$78.67±2.52^{cd}$
BC-11	$3.11±0.41^{def}$	$5.02±0.79^{cd}$	$438.67±3.06^{bcd}$	$66.00±3.61^{cde}$	$86.00±2.65^{ab}$
BC-12	$3.70±0.53^{a}$	$5.14±0.38^{c}$	$440.67±9.29^{bc}$	$66.33±4.73^{cde}$	$80.33±3.79^{bcd}$
BC-13	$3.74±0.42^{a}$	$6.14±1.01^{b}$	$458.33±24.83^{b}$	$75.00±3.00^{b}$	$85.67±1.53^{ab}$
BC-14	$3.60±0.57^{ab}$	$7.44±0.58^{a}$	$630.67±21.55^{a}$	$87.33±2.08^{a}$	$87.67±0.58^{a}$
BC-15	$3.38±0.36^{abcd}$	$5.86±0.74^{b}$	$431.33±19.55^{bcd}$	$69.67±2.52^{bc}$	$84.33±1.53^{abc}$
BC-16	$3.07±0.45^{def}$	$4.98±0.46^{cde}$	$436.00±15.00^{bcd}$	$63.00±4.58^{de}$	$79.00±3.61^{cd}$
BC-17	$2.93±0.42^{efg}$	$4.73±0.63^{cde}$	$427.67±13.05^{cd}$	$63.67±3.21^{cde}$	$78.00±1.00^{d}$
BC-18	$2.77±0.45^{fgh}$	$4.52±0.44^{def}$	$411.00±8.19^{de}$	$63.67±3.79^{cde}$	$79.33±5.13^{cd}$
BC-19	$3.12±0.52^{def}$	$4.81±0.58^{cde}$	$426.33±5.03^{cd}$	$61.33±3.51^{def}$	$80.00±1.00^{bcd}$

Table S2 Genome properties of *Cronobacter dublinensis* BC-14

Feature	*C. dublinensis* BC-14
Genome size (bp)	4,491,274
GC content (%)	58.09
Total number of genes	4236
Number of CDSs	4058
tRNA genes	71
Genomic islands	4
Genes allocated to Refseq	4021
Genes allocated to KEGG	2806
Genes allocated to Pfam	3705
Genes allocated to GO	2637
Genes allocated to COG	3458

Table S3 Functional genes associated with plant growth and drought resistance for *Cronobacter dublinensis* BC-14.

Function	Gene		Start	End	Strand	scafold
IAA production	trpA	tryptophan synthase alpha chain	88599	89408	-	contig 000001
	trpB	tryptophan synthase beta chain	89408	90601	-	contig 000001
	trpC	indole-3-glycerol phosphate synthase	90613	91971	-	contig 000001
	trpD	anthranilate phosphoribosyltransferase	91975	93570	-	contig 000001
	trpE	anthranilate synthase component I	93570	95132	-	contig 000001
	trpS	Tryptophanyl-tRNA synthetase	332342	333346	+	contig 000001
	ipdC	indolepyruvate decarboxylase	186286	187953	+	contig 000001
Phosphate solubilization	phoB	phosphate regulon transcriptional regul atory protein	1500751	1501440	-	contig 000001
	phoH	phosphate starvation-inducible protein	887663	888451	-	contig 000002
	phoR	two-component sensor histidine kinase	1499415	1500728	-	contig 000001
	phoU	phosphate transport system protein	35180	35905	+	contig 000001
	phoP	two-component system ompR family response regulator	789369	790040	+	contig 000001
	phoQ	two-component system ompR family response regulator	789369	790040	+	contig 000007
	pstS	phosphate transport system substrate-binding protein	31268	32308	+	contig 000001
	pstC	phosphate transport system permease protein	32448	33407	+	contig 000001
	phoB	Phosphate regulon transcriptional regulatory protein	1500751	1501440	-	contig 000007
	pstA	phosphate transport system permease protein	33407	34297	+	contig 000007
	pstB	phosphate transport system ATP-binding protein	34348	35121	+	contig 000001
Siderophore production	entC	isochorismate synthase	136673	137839	+	contig 000007
	dhbE	2,3-dihydroxybenzoate-AMP ligase	137849	139453	+	contig 000002
	entD	4'-phosphopantetheinyl transferase	1293733	1294428	+	contig 000002
	entA	2,3-dihydro-2,3-dihydroxybenzoate dehydrogenase	140329	141081	+	contig 000001
	entF	L-serine- [L-seryl-carrier protein] ligase	1285806	1289700	-	contig 000002
	fhuD	ferric hydroxamate transport system substrate-binding protein	466197	467093	+	contig 000001
	fhuB	ferric hydroxamate transport system permease protein	467090	469075	+	contig 000004
	fhuC	ferric hydroxamate transport system ATP-binding protein	465400	466197	+	contig 000004
Root colonization	flgH	Flagellar L-ring protein	844987	845694	-	contig 000004

	flgL	flagellar hook-associated protein	840201	841163	-	contig 000001
	flgK	flagellar hook-associated protein	841178	842836	-	contig 000001
	flgI	flagellar P-ring protein	843877	844974	-	contig 000001
	flgG	flagellar basal-body rod protein	845743	846525	-	contig 000001
	flgF	flagellar basal-body rod protein	846542	847300	-	contig 000001
	flgE	flagellar hook protein	847321	848553	-	contig 000001
	flgD	flagellar basal-body rod modification protein	848580	849260	-	contig 000001
	flgC	flagellar basal-body rod protein	849272	849676	-	contig 000001
	flgB	flagellar basal-body rod protein	849690	850103	-	contig 000001
	flgM	negative regulator of flagellin synthesis	851013	851306	+	contig 000001
	flgA	flagellar basal body P-ring formation protein	850257	850916	+	contig 000001
	flgN	flagellar biosynthesis protein	851310	851735	+	contig 000001
	cheR	chemotaxis protein methyltransferase(EC 2.1.1.80)	652169	653035	+	contig 000002
	cheB	two-component system chemotaxis family protein-glutamate methylesterase	653032	654081	+	contig 000002
	cheY	two-component system chemotaxis family chemotaxis protein	654099	654488	+	contig 000002
	cheZ	chemotaxis protein	654499	655140	+	contig 000002
	cheW	purine-binding chemotaxis protein	643849	644352	+	contig 000002
	cheA	two-component system chemotaxis family sensor kinase	641802	643820	+	contig 000002
Oxidative stress	sodC	Cu/Zn superoxide dismutase	543630	544148	-	contig 000001
	sodA	Superoxide dismutase	49819	50439	+	contig 000008
	cotJC	Mn-containing catalase	411831	412736	+	contig 000001
	katG	catalase-peroxidase	312627	314807	+	contig 000003
	tpx	Peroxiredoxin	196894	197400		contig 000001
	yciW1	peroxiredoxin activity	492080	493174	+	contig 000001
	yurZ	peroxiredoxin activity	765161	765883	-	contig 000001
	pcaC	peroxiredoxin activity	45439	45816	+	contig 000002
	bcp	thioredoxin-dependent peroxiredoxin	108089	108559	-	contig 000002
	yciW	peroxiredoxin activity	781856	782335	+	contig 000002
	osmC	lipoyl-dependent peroxiredoxin	251007	251435	-	contig 000004

	ahpF	alkyl hydroperoxide reductase subunit F	1278278	1279843	-	contig 000001
	trxB	thioredoxin-disulfide reductase	1010329	1011297	+	contig 000001
Spermidine	gsp	glutathionylspermidine synthase preATP-grasp	138205	139392	+	contig 000003
	speE	Spermidine synthase	439324	440190	-	contig 000004
Trehalose	treF	Trehalase	352505	354145	-	contig 000001
	treC	trehalose-6-phosphate hydrolase	201384	203039	-	contig 000003
Cold active chaperones	cspC	cold shock protein	760866	761078	-	contig 000001

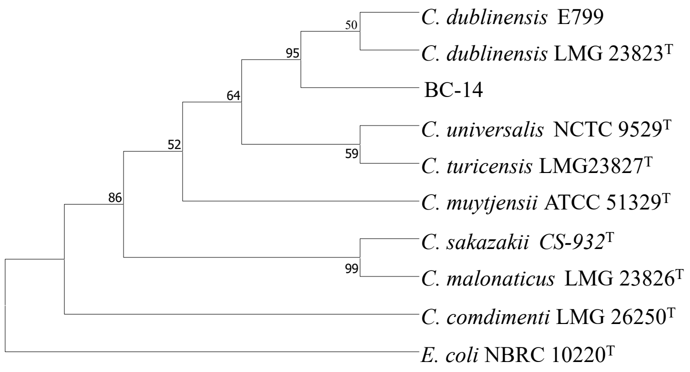

Figure S1 The phylogenetic tree based on 16S rRNA using the maximum likelihood method. Bootstrap values (>60%) from 1000 replicates are shown at nodes. E. coli NBRC 10220 was selected as the outgroup. The present strains are marked in bold. ^{T}= type strain

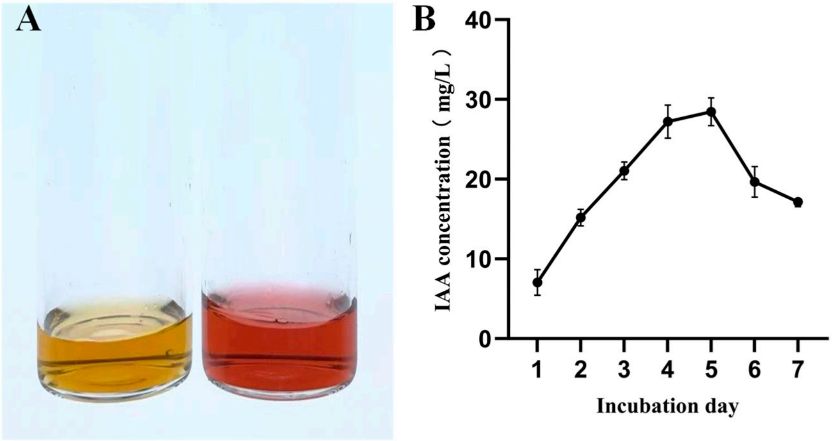

Figure S2 Detection of the IAA production in *Cronobacter dublinensis* BC-14. (A) Salkowski test of the negative control (left) and the *Cronobacter dublinensis* BC-14 broth (right). (B) The IAA concentration in different incubation times.

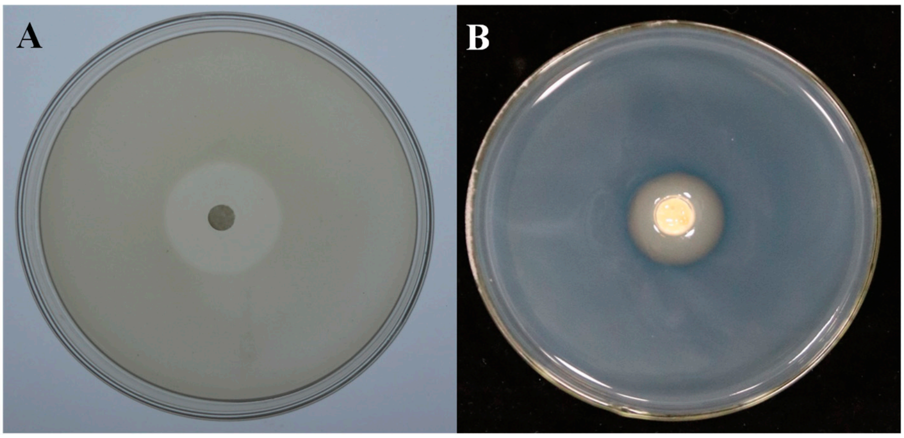

Figure S3 Clear zone formation around the colony in inorganic phosphorus agar medium indicated the ability to solubilize inorganic phosphorus(A); Yellow-orange halos in CAS agar medium indicated siderophore production(B).
